# Emerging roles of cancer-testis antigenes, semenogelin 1 and 2, in neoplastic cells

**DOI:** 10.1038/s41420-021-00482-4

**Published:** 2021-05-08

**Authors:** Oleg Shuvalov, Alyona Kizenko, Alexey Petukhov, Olga Fedorova, Alexandra Daks, Nikolai Barlev

**Affiliations:** 1 Institute of cytology RAS, St-Petersburg, Russia; 2 Almazov Federal North-West Medical Research Centre, St-Petersburg, Russia; 3 MIPT, Dolgoprudny, Moscow region, Moscow, Russia

**Keywords:** Cancer models, Oncogene proteins

## Abstract

Cancer-testicular Antigens (CTAs) belong to a group of proteins that under normal conditions are strictly expressed in a male’s reproductive tissues. However, upon malignisation, they are frequently re-expressed in neoplastic tissues of various origin. A number of studies have shown that different CTAs affect growth, migration and invasion of tumor cells and favor cancer development and metastasis. Two members of the CTA group, Semenogelin 1 and 2 (SEMG1 and SEMG2, or SEMGs) represent the major component of human seminal fluid. They regulate the motility and capacitation of sperm. They are often re-expressed in different malignancies including breast cancer. However, there is almost no information about the functional properties of SEMGs in cancer cells. In this review, we highlight the role of SEMGs in the reproductive system and also summarize the data on their expression and functions in malignant cells of various origins.

## Facts

- Cancer-testis Antigens (CTAs) are frequently expressed in different neoplasms, induce EMT and are associated with unfavorable prognosis for patient’s survival
- SEMG1 and SEMG2 are two non-X linked CTAs that normally regulate motility and maturation of sperm but become re-expressed in different cancerous tissues
- SEMG1 and SEMG2 possess biological activity in tumors, can both increase energy metabolism and display anti-proliferative properties

## Open Questions

- Do SEMG1 and SEMG2 undergo processing and do their functions in cancer cells depend on proteases?
- What are molecular functions of SEMG1 and SEMG2 in tumor cells with different background?
- Do SEMG1 and SEMG2 elicit immune response in cancer patients?
- Is it possible to use them as tumor’s biomarkers?

## Introduction

Cancer-testicular antigens (CTAs) have long been considered as potential prognostic tumor markers^1^ and as targets for the immunotherapy of malignant tumors^2^. CTAs are primarily expressed in germ cells of the male’s body. Moreover, they are often expressed in malignant neoplasms of various origins, and therefore they are referred to as tumor antigens. Thus, many CTAs are immunogenic markers of tumor cells, which makes them valuable targets for antitumor vaccines and therapy using reprogrammed T-cells (chimeric antigen receptors)^3^.

Semenogelin 1 and 2 (SEMG1 and SEMG2, respectively) are two autosomal CTAs. They are the major proteins of human semen and are mainly expressed by the glandular epithelium of seminal vesicles^4^. Several of their functions are well described: the regulation of sperm motility and capacitation, as well as an antibacterial protection of the former.

Surprisingly, although the SEMGs genes share a high degree of homology (78%) between them, almost all the information available in literature about their biological role in reproduction concerns only SEMG1. Interestingly, it was shown that in addition to the tissues of reproductive organs, SEMGs can also be detected at the mRNA level in a number of other tissues—the retina, epithelium of the gastrointestinal tract, skeletal muscle, central nervous system tissues^5^, etc. In line with their belonging to a group of CTAs, SEMGs were found expressed in various human malignancies. So far, there is no data available on the functional role of SEMGs in neoplastic cells, except in prostate cancer.

Below we summarize the information on known functions of SEMGs in reproductive tissues, as well as data on their expression in other normal tissues and malignant neoplasms of various origins. Based on generalizations, we analyze the possible functions of SEMGs in neoplastic cells.

### Cancer-testicular antigens

Tumor cells of various origins exhibit on their surface, and excrete into the bloodstream, various antigens that can be recognized by immune system, followed by the eradication of such cells. In this way, the specific antitumor immune response can be generated.

This underlies the development of antitumor vaccines aimed at the activation of specific antitumor immunity in patients. Moreover, the immune response to tumor antigens varies and is often not effective due to the presence of a number of protective mechanisms in the tumors, allowing them to escape from the immune response^6^.

Tumor antigens can be divided into two groups: tumor-specific and tumor-associated antigens. Tumor-specific antigens are unique antigens that are characteristic only of neoplastic cells^7^. Such antigens, for example, include the well-known chimeric protein BCR-ABL, which is a specific marker of acute myeloid leukemia cells.

Tumor-associated antigens are the most widely represented tumor antigens. They include proteins, which are often present on the surface of tumor cells but are also expressed by normal cells of some tissues. One of the tumor-associated antigen subgroups is cancer-testicular antigens (CTAs), which include proteins synthesized primarily in the tissues of the testis (spermatogonia, spermatids, spermatozoa), as well as in the trophoblast and placenta^8^. In addition, the expression of these proteins is reported in neoplastic tissues of various origins^9^. Their presence is often associated with low-grade tumors and an unfavorable prognosis for the patient’s survival^10,11^.

The main features according to which antigens are assigned to the CTAs group are the predominant expression of corresponding genes in the germ cells of the testis, their frequent expression in neoplastic cells, and the frequent localization at the X chromosome^12^. Thus, proteins can be classified as CTAs when their expression in normal is observed in germ cells but also can also be re-expressed in tumors. However, not all of these proteins are capable of eliciting immune response; nonetheless they are united by the term ‘CTAs’.

Currently, the CTAs database^13^ (http://www.cta.lncc.br/) contains information about 204 genes from more than 100 families; the level of mRNA, protein expression and their immunogenicity. CTAs can be divided into two sub-groups: ones localized at the X chromosome (X-linked CTAs or X-CTAs) and distributed throughout the rest of the genome (non-X-CTAs). X-CTAs coding genes are characterized by multiple copies; together they occupy about 10% of the X chromosome. X-CTAs make up about half of all CTAs and, as a rule, form clusters at the chromosome^14^. They often form multi-gene families. On the contrary, non-X-CTAs coding genes are distributed throughout the genome and are most often represented by single-copy genes. X-CTAs are most often expressed in spermatogonia, while non-X-CTAs are predominantly expressed in the later stages of germ cell differentiation in spermatocytes^8^.

**Fig. 1:**
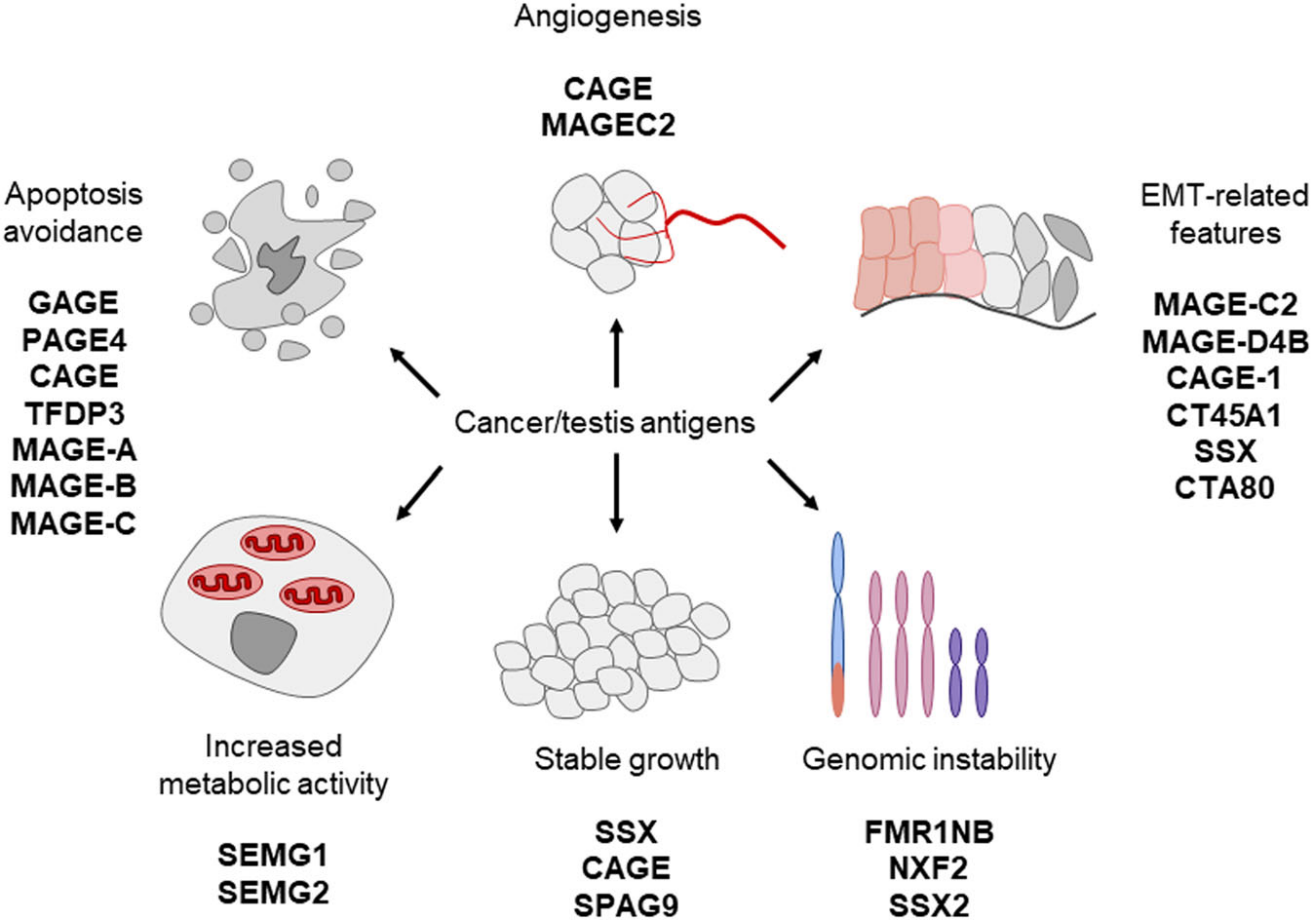
Known functions of cancer/testis antigens in neoplastic tissues. Explanations are given in the text.

CTAs are known to have different biological properties in neoplastic cells (Fig. 1). It was shown that CTAs can provide sustained tumor growth, tissue invasion and metastasis, genomic instability, evading of apoptosis, inducing angiogenesis and so on (for comprehensive review see please^2,15,16^.

### Cancer-testicular antigens affect EMT

The epithelial-to-mesenchymal transition (EMT) is the process of acquiring mesenchymal phenotype by epithelial cells. In normal, this process occurs during embryonic development, tissue regeneration, organ fibrosis, and wound healing^17^. However, in cancer, EMT is associated with metastasis formation. By undergoing EMT, cancer cells disrupt cell-cell adhesion, polarity and acquire migratory and invasive properties^18^. These EMT-associated events allow cancer cells to invade the bloodstream, followed by dissemination throughout the body^19^.

However, EMT is also reversible by the mesenchymal-to-epithelial transition (MET). It is deemed to affect circulating cancer cells when they reach a desirable metastatic niche to develop secondary tumors. EMT is not only associated with the development of metastasis but also contributes to the increased resistance of neoplastic cells against multiple chemotherapeutics and radiotherapy^20,21^.

Since metastasis is the main reason for cancer-mediated cell death, attention of many scientists is focused on the study of mechanisms regulating EMT. Indeed, various studies have shown that CTAs both trigger EMT and contribute to the genesis of cancer stem-like cells, escalating tumorigenesis, invasion, and metastasis^22^. Some of these studies listed in Table 1 show that well-known CTAs induce EMT, enhance migration and evasion of cancer cells in different models of breast, gastric, colon, hepatocellular, ovarian, cervical cancer, melanoma, and multiple myeloma.

**Table 1:** CTAs promote EMT in neoplasms of different origin.

Gene	Type of cancer	Effect on EMT and EMT-related processes?	Reference
SPANXB1	Triple Negative Breast Cancer (TNBC)	Promotes migration, invasion and reactive oxygen species production of TNBC cells	^26^
SPANX-A/C/D and CTAG2	Breast cancer	CTAG2 is necessary for directional migration SPANX-A/C/D is required for formation of actin-rich cellular protrusions that reorganize the extracellular matrix	^27^
TSP50	Gastric cancer	Promotes the proliferation, migration and invasion of gastric cancer cells involving NF-κB dependent EMT activation	^28^
CT45A1	Breast cancer	Constitutively activates ERK and CREB signaling pathways, promotes EMT, and increases cell stemness, tumorigenesis, invasion, and metastasis	^29^
MAGEC2	Hepatocellular carcinoma	Promotes EMT	^30^
MAGEC2	Breast cancer	Promotes EMT by binding to a transcriptional repressor, KAP1, which plays a critical role in promoting cadherin switching and acquiring mesenchymal cell molecular patterns	^23^
SPAG9	Ovarian cancer	Increased the expression of mesenchymal markers and decreased expression of epithelial markers	^31^
SPAG9	Bladder cancer	Promotes migration, decreases E-cadherin and increases Vimentin	^32^
PIWIL1 (HIWI)	Hepatocellular carcinoma	Promotes cell migration and invasion	^33^
PIWIL2 (HILI)	Colon cancer	Activates MMP9 and enhance migration and invasion	^34^
SSX	Melanoma	Activates MAPK/Erk and Wnt signaling, promotes EMT	^35^

Although a number of studies point to the CTAs-induced augmentation of mesenchymal markers (vimentin, N-cadherin, Snail, Slug, etc.), simultaneously with a decrease of epithelial marker E-cadherin, there is only one publication that demonstrates a direct mechanism by which one of the CTAs, MAGEC2, affects EMT in breast cancer. This mechanism is realized by MAGEC2 binding to the KAP1 transcriptional repressor, which plays a critical role in promoting the cadherins switch and hence acquiring mesenchymal features by cancer cells^23^. However, a number of other published works listed in Table 1 strongly suggest that the CTAs affect EMT-related processes —migration and invasion when they are accompanied by loss of epithelial and the acquisition of mesenchymal markers.

It should be noted however, that there are reports about CTAs-mediated inhibition of EMT, migration and invasion capabilities of some cancer models^24,25^,. This suggests that the impact of certain CTAs on EMT can depend on the particular cellular context.

### SEMG1 and SEMG2: structure and known functions in reproduction

SEMG1 and SEMG2 are the main proteins of human seminal fluid, which together with fibronectin create the gel-like structure of the ejaculated semen and make up 20–40% of its volume^4,26^. They are synthesized and secreted mainly by the glandular epithelium of the seminal vesicles. There is evidence that transcripts of SEMGs are also detected in epididymis, vas deferens and prostate, but the functional role of these proteins in these organs is not known today.

**Fig. 2:**
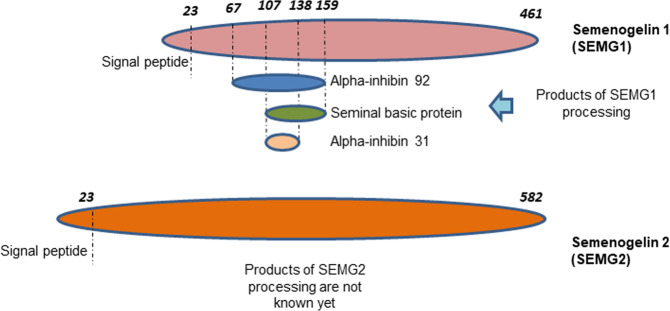
A scheme of semenogelin 1 (a) and semenogelin 2 (b) protein structure and their main functional degradation products taking part in reproduction. Dotted lines show PSA (prostate-specific antigen) cleavage sites. Explanations are given in the text.

Gene encoding SEMGs are localized at the long arm of the 20th chromosome and contain 3 exons. The first exon encodes a signal peptide, the second—the main part of the protein, the third—3’-untranslated region. While the first and third exons of SEMG1 and 2 have conserved sequences, the second exons differ in structure. SEMGs have 78% homology in protein level. SEMG1 is synthesized as a precursor of 461 a. a. (Fig. 2) and is further processed up to 439 a.a. by cleavage of hydrophobic amino-terminal signal peptide. Thus, the secreted SEMG1 has a molecular weight of 52 kDa. In turn, SEMG2 is also synthesized as a precursor of 582 a. a. in size (Fig. 1) followed by cleavage of the amino-terminal signal peptide^27,28^,. Secreted SEMG2 consists of 559 a.a. and can be 71 or 76 kDa in size. 71 kDa SEMG2 corresponds to the native protein, while half of all secreted SEMG2 has N-glycosylated asparagine at position 272 (Fig. 2) which corresponds to a molecular weight of 76 kDa^27^.

SEMGs and products of their proteolysis perform a number of important functions in seminal fluid. In particular, they regulate the motility^4,29^, and capacitation of spermatozoa^30^, and provide sperm with antibacterial protection^23^. Although SEMG1 and 2 are highly homologous, almost all information available in literature on the role of SEMGs in reproductive processes concern only SEMG1. The functional role of SEMG2 remains poorly studied even in the reproductive process.

Semenal fluid is a mixture of the seminal vesicle’s secretion, and the secretion of prostate and sperm coming from the epididymis^31^. The main function of SEMG1 is to ensure the coagulation of spermatozoa—the compaction of seminal fluid immediately after ejaculation. Then, over time, it gradually liquefies. The coagulation prevents the loss of sperm in the vagina, and its subsequent liquefaction provides sperm the ability move towards the cervix^4^.

During ejaculation, SEMGs and to a lesser extent fibronectin, form a matrix due to non-covalent interactions and disulfide bridges that impede the movement of sperm cells to the ovum^32,33^. Thus, native SEMG1 inhibits sperm motility. Over the next 5–15 min, the proteolysis of SEMG1 occurs followed by liquefaction of the ejaculate and the release of sperm, which then rush to the ovum. Proteolysis of SEMGs is carried out mainly by serine protease, a prostate-specific antigen (PSA), which is part of the secretion of the prostate gland and has chymotrypsin-like proteolytic activity^29^.

To date, three functional peptides have been identified (Fig. 2) obtained as a result of the proteolytic degradation of SEMG1: alpha-inhibin-92, alpha-inhibin-31^34^ and the base protein of seminal fluid^35^. Using a mouse model, it was shown that alpha-inhibin-92 and alpha-inhibin-31 inhibit the secretion of pituitary follicle-stimulating hormone^34^. The data available in literature indicates that SEMG2 is also a substrate for PSA and, accordingly, undergoes fragmentation^27,36^. However, the corresponding peptides resulting from the PSA-dependent cleavage of SEMG2 are not currently described.

**Fig. 3:**
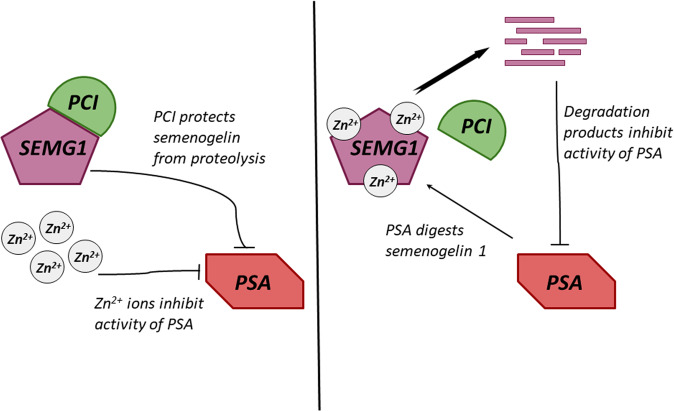
Regulation of the semenogelin 1 cleavage in seminal vesicles (a) and after ejaculation (b). SEMG1—semenogelin 1, PSA—prostate-specific antigen, PAP—prostatic acid phosphatase, PCI—protein C inhibitor. Explanations are given in the text.

The degradation of SEMG1 is regulated by the PCI protein (protein C inhibitor) (Fig. 3), which, in addition to SEMG1, is also associated with PSA^37^. SEMG1 has the same affinity for both PCI and PSA, and the formation of the corresponding protein complexes depends on the pH, ionic strength, the presence of heparin-like proteins, negatively charged dextran sulfate and divalent cations (in particular zinc). The PCI-SEMG1 complex occurs in the seminal vesicles before ejaculation (Fig. 3), which prevents the proteolytic degradation of SEMG1. During ejaculation, as seminal vesicle secretions move along the ducts, PCI loses the ability to interact with SEMG1 and binds PSA, which, in turn, acquires proteolytitivity and cleaves the SEMG1 into functional oligopeptides (Fig. 3). The PCI–SEMG1 complex is formed at low concentrations of zinc ions in seminal vesicles (0.86 mM), and then dissociates in seminal fluid due to the increase in the concentration of zinc ions (6.2 mM)^4^.

The proteolytic activity of PSA in the seminal fluid is inhibited by zinc ions, while SEMG1is able to chelate these ions (Fig. 3). After ejaculation and SEMG1-mediated zinc binding, PSA activates and cleaves SEMG1 into functional oligopeptides^4^. It was also shown that PCI can form complexes with PSA, thereby most likely, decreasing its proteolytic activity. However, the proportion of such complexes in the seminal fluid is small.

Another important factor of SEMGs-mediated regulation of sperm motility is a serine protease inhibitor Eppin. It is synthesized and excreted by Sertoli cells, as well as epithelial cells of epididymis^38^. Eppin interacts with a protein complex located on the surface of sperm. This complex consists of lactotransferrnin, clusterin, SEMG1 and, accordingly, Eppin^39^. It has been shown that Eppin acts as receptor for SEMG1 and their interaction is extremely important for SG-mediated inhibition of sperm motility. In this case, Eppin regulates the degradation of SEMG1 due to the negative effect on PSA activity. In addition, it was shown that the interaction of SEMG1 with Eppin on the surface of spermatozoa leads to a decrease in intracellular pH and the absence of oscillations of intracellular calcium, which ultimately hinders sperm motility^38^.

Thus, negative regulation of sperm motility is carried out by both the participation of SEMG1 in the formation of a matrix that impedes their motility, as well as by means of signal cascades unknown so far, initiated after interaction with Eppin and leading to a change in intracellular pH and calcium waves. Apparently, another function of SEMG1 is the suppression of premature сapacitation^27^. Capacitation is the process of sperm maturation in the female genital tract when sperm acquires the ability to penetrate the ovum. It was shown that SEMG1 leads to inhibition of the fertilizing ability of sperm due to the suppression of reactive oxygen species, the generation of which is extremely important for capacitation. After getting into the female’s genital tract, SEMG1 processing occurs and capacitation becomes possible^30^.

In addition, in vitro experiments have shown that purified SEMG1 and 2 contribute to the activation of testicular hyaluronidase in bovines and humans^40^. Hyaluronidase is secreted by the sperm acrosome in order to hydrolyze hyaluronan, which constitutes a significant part of the intercellular matrix. Thus, activation of hyaluronidase promotes gamete fusion. However, the role of SG in this process in vivo remains unstudied.

Another function of SEMG1 is the antibacterial protection of sperm. SEMG1 itself has antibacterial activity^23^, however products of its processing mediated by PSA^41^ are characterized by significantly more pronounced antimicrobial activity against various bacterial strains.

Thus, SEMGs participate in reproductive function of the body by regulating the motility and maturation of sperm and its protection from bacteria.

### Expression of SEMGs in normal tissues and in tumors

As mentioned above, SEMGs are expressed in germinal tissues. However, a number of research groups have shown their presence at both mRNA and protein levels in several normal tissues^5,42^. In particular, transcripts of SEMGs genes were found in seminal vesicles, seminal ducts, prostate, appendages, trachea, salivary and mammary glands, as well as skin^5^. In this case, the organs of the gastric tract predominantly expressed SEMG1, while in kidneys and testicle the transcript encoding SEMG2 was mainly detected. In addition, mRNAs of SEMGs were detected in skeletal muscle, retina and in the tissues of the central nervous system^5^. Apparently, SEMGs expression is not limited to epithelial tissues and all three germ layers are able to express them.

By using immunohistochemistry, it was shown that prostate tumors more often express both SEMGs than healthy surrounding tissues^41^. In this case, an inverse correlation between SEMG2 expression and the tumor score on the Gleason scale is noted, which indicates the association of SEMG2 with more differentiated tumors. At the same time, in accordance with the Kaplan-Meyer survival curves, the combination of high levels of SEMG1 expression and low levels of SEMG2 is associated with a low survival of patients with prostate tumors^43^.

Predominantly nuclear localization of SEMG1 has been shown in prostate carcinomas in comparison with surrounding healthy tissues^44^. Other researchers have shown that SEMG1 promotes the growth of androgen-sensitive prostate cancer cells, being a zinc-dependent co-activator of the androgen receptor^45^. In addition, SEMG1 protects prostate tumor cells from zinc-mediated cytotoxicity. Thus, literature data indicates the potential difference in the functions of SEMG1 and SEMG2 in prostate cancer.

However, there is evidence of a positive association between SEMG1 level and a patient’s survival in case of renal carcinoma. In this case, SEMG1 expression was significantly more often detected in malignant tumors when compared with benign and corresponding normal tissues^46^. In addition, an inverse association was observed between the expression of SEMG1 and the stage of tumor development. According to the Kaplan–Meier survival curves, a low level of SEMG1 was associated with poor patient outcomes^46^. Thus, these data indicate the oncosuppressive properties of SEMG1 in the case of renal carcinomas.

It has been shown that SEMG1 is expressed by tumor cells in chronic myeloid leukemia (CML), chronic lymphoblastic leukemia (CLL) and myeloma^47^. Moreover, the expression of SEMG1 in myeloma cells was increased in the presence of IL-4 and IL-6^48^. SEMG1 and SEMG2 were detected in a number of cell lines of human lung cancer and melanoma^49^. Immunohistochemistry has shown the expression of SEMGs in 12 of 13 samples of squamous cell lung carcinoma and adenocarcinoma. In addition, SEMGs can also be detected in the blood serum of patients with non-small cell lung cancer^50^ which indicates the possibility of their potential use as biomarkers for the diagnosis of cancer.

### Potential biological functions of SEMGs in malignant cells

As mentioned above, the functions of SEMGs (mainly SEMG1) are known only in reproduction, and information on the functions of SEMGs in malignant neoplasms is particularly absent. An exception is the only study in which SEMG1 was shown as co-activator of the androgen receptor in prostate cancer which protects cancer cells from zinc-mediated cytotoxicity^46^.

However, the association between levels of SEMGs’ expression and patient survival can differ depending on the particular tumor type and can be contradictory even in tumors of the same origin. As mentioned above, the expression of SEMG1 in prostate cancer can be associated with both positive and negative patient survival^43–46^. Moreover, SEMG1 and SEMG2 can be contrary associated with the patient’s outcome in prostate cancer^43^. It is possible to suggest that SEMG1 and SEMG2 may have different functions even in the same cells. This suggestion is supported by our data about the different spectrum of SEMGs’ interactants in MCF7 breast carcinoma cell model identified by LC-MS/MS (Shuvalov et al., in press). In the same research, we have also shown that SEMGs can up-regulate energy metabolism of cancer cell models by increasing glycolysis and respiration.

Zhang with colleagues^46^ have shown a positive association between the level of SEMG1 and a patient’s survival in the case of renal carcinoma. We have also demonstrated that SEMGs possess different anti-proliferative properties of lung adenocarcinoma H1299 cells^51^. Apparently, SEMGs can possess both oncogenic and oncosupressive features depending on the particular cellular background.

Since SEMGs belong to CTAs and are normally expressed primarily in germ cells, the legitimate question is whether SEMGs perform any functions in tumor cells or they can be just cancer bystanders, i.e. genes whose expression is restored in cancer cells as part of the larger embryonic genes signature re-activation program. It is well known that tumors, especially the low-differentiated ones, often express a gene pattern similar to embryonic stem cells^52–54^. Germ cell precursors, like trophoblast cells have much in common with tumor cells^2^. For example, in the process of colonization of gonad primordia, germ cells are very mobile and have the ability to invade tissues^55^. During spermatogenesis, spermatogonia maintain proliferative activity throughout their life. In turn, trophoblast cells are able to invade the endometrium and actively proliferate, forming part of the placenta. All these are very similar to properties of tumor cells. Thus, neoplastic cells have much in common with germ cells^53,54^ and from this point of view it is possible to explain why they express a similar gene pattern.

Taking into account our data about SEMGs’ features in several cancer cell models, literature data about biological activity of SEMG1 in prostate cancer and the known biological role of SEMGs in reproduction, we hypothesize that SEMGs expression can have an impact on malignant cells. Also, it is interesting to note that the biological role for a number of CTAs in tumor cells has been described. In particular, CTAs can promote proliferation (SSX2, CAGE), genomic instability (FMR1NB, NXF2, SSX2), evasion from apoptosis (MAGE-A, MAGE-B, MAGE-C, CAGE, PAGE4), induction of angiogenesis (CAGE), invasion and metastasis (MAGE-C2)^2^.

For example, there are two well-known oncofetal proteins, Carcinoembryonic antigen (CEA) and alpha-fetoprotein (AFP), which originate within tumor cells and enter the bloodstream either by secretion from the tumor or as a breakdown product of tumor cells. Normally they are expressed during embryogenesis and then become epigenetically silenced. Importantly, their expression may be re-activated in certain cancers making them potentially useful tumor markers. Furthermore, there are evidences demonstrating the active roles in carcinogenesis for both of them^56^_._ AFP was shown to function as a growth regulator by binding to key proteins involved in signaling pathways. In particular, AFP has been demonstrated to block the RA-RAR signaling to disrupt the forward transmission of apoptotic signaling^57^^,^^58^ AFP also interacts with PTEN to activate the PI3K/AKT pathway, leading to aberrant growth and migration of HCC cells^49,59–61^. Furthermore, AFP inhibits autophagy to promote malignant behavior in hepatocellular carcinoma cells by activating PI3K/AKT/mTOR signaling^62^. Tumor-derived AFP impairs the differentiation and T-cell stimulatory activity of human dendritic cells^63^. Taking all these facts together, it would be fair to say that at least some of the re-activated CTAs play active roles in tumorigenesis.

As discussed above, SEMGs regulate the motility of sperm (create a gel-like matrix, activate hyaluronase), regulate its maturation (are scavengers of reactive oxygen species during capacitation) and participate in zinc homeostasis. These functions can also occur in neoplastic cells. For instance, the motility of a tumor cell is the basis of invasion and metastasis^64^. At the same time, rapidly proliferating cancer cells are under constitutive oxidative stress^65^. Moreover, zinc is capable of exerting a strong toxic effect on tumor cells of various origins in comparison with normal cells; in addition, the concentration of zinc ions in tumor tissues is often reduced^66^. Thus, the already known functions of SEMGs in reproduction can be used by tumor cells to maintain their vital functions. However, each of these potential molecular mechanisms requires a separate study.

In addition, SEMGs can possess some other molecular functions in cancer cells unknown yet. According to the BioGrid database (https://thebiogrid.org/), SEMGs interact with more than twenty different proteins, among which there are regulators of the cell cycle, apoptosis, etc. The functional role of their interaction with SEMGs remains to be investigated.

### Conclusion and perspectives

SEMGs are CTAs which are expressed in malignancies of various origins. However, their biological functions were studied exclusively in the context of the reproductive function of the body. So, this is important to ask about the functions of SEMGs in malignant cells.

Do SEMGs undergo processing and do their functions in cancer cells depend on proteases? What are molecular and phenotypic changes in tumor cells during SEMGs expression or silencing? What are the molecular mechanisms mediating this effect? Answers to these questions will help clarify the functional role of SEMGs in oncogenesis.

Also, questions about the immunogenicity of SEMGs and their possible role in the antitumor immune response are extremely important. It has been shown that in a number of cases of CML and CLL expressing SEMG1, antibodies specific to SEMG1 were detected in patients^47^. It means that in patients with tumor expressing SEMGs, the immune response against these proteins may occur, which contributes to the destruction of tumor cells by T-lymphocytes.

However, in another work^67^ it was shown that SEMG1 can inhibit the proliferation of peripheral blood lymphocytes and the production of immunoglobulins. Thus, SEMGs can potentially be involved in the antitumor immune response. So, the assessment of SEMGs expression in tumors and their occurrence in the serum of cancer patients, as well as the study of their functions can be of clinical significance in the context of identifying new tumor markers and the development of new approaches to antitumor immunotherapy.
